# Early and Long-Term Outcomes after On-Pump and Off-Pump Coronary-Artery Bypass Grafting in Patients with Severe Left Ventricular Dysfunction and a Giant Left Ventricle

**DOI:** 10.3390/jcdd9090298

**Published:** 2022-09-06

**Authors:** Chen Wang, Yefan Jiang, Qingpeng Wang, Rui Tian, Dashuai Wang, Xionggang Jiang, Nianguo Dong, Si Chen, Xinzhong Chen

**Affiliations:** 1 Department of Cardiovascular Surgery, Union Hospital, Tongji Medical College, Huazhong University of Science and Technology, Wuhan 430000, China; 2 Department of Cardiovascular Surgery, The First Affiliated Hospital of Nanjing Medical University, Nanjing 210000, China

**Keywords:** coronary artery bypass grafting, severe left ventricular dysfunction, giant left ventricle, on-pump, off-pump

## Abstract

Introduction: No previous studies comparing the outcomes between off-pump coronary artery bypass grafting (off-pump CABG, OPCAB) and on-pump CABG (ONCAB) have been performed in patients with severe left ventricular dysfunction (LVD) and a giant left ventricle. We aimed to investigate whether such patients could benefit from OPCAB. Methods: From January 2011 to January 2021, a total of 98 patients with severe LVD and a giant left ventricle underwent isolated CABG (ONCAB 46, OPCAB 52) in Wuhan Union Hospital. The clinical data were collected retrospectively and propensity score matching was performed to adjust baseline characteristics. Results: After propensity matching, the two groups were comparable in baseline variables. The OPCAB group had a higher rate of incomplete revascularization than the ONCAB group (25.0% vs. 9.1%; *p* = 0.047). The 30-day mortality was similar between the matched groups (4.5% vs. 4.5%; *p* = 1.000) but the OPCAB group had a lower risk of postoperative IABP usage (9.1% vs. 25.0%; *p* = 0.047) and renal insufficiency (11.4% vs. 29.5%; *p* = 0.034). The long-term probability of survival (log-rank test, *p* = 0.450) was similar between the two groups but the OPCAB group had a lower probability of major adverse cardiovascular events (log-rank test, *p* = 0.038). Conclusions: For patients with severe LVD and a giant left ventricle, OPCAB reduced early postoperative complications while sacrificing long-term quality of life compared to those having ONCAB.

## 1. Introduction

Patients with severe left ventricular dysfunction (LVD) and coronary artery disease (CAD) are challenges for clinicians. Coronary artery bypass grafting (CABG) has been proven to be an effective treatment for these patients, superior to medical therapy and percutaneous coronary intervention (PCI); it not only relieves symptoms but prolongs survival [1,2,3]. In patients with multivessel coronary disease and severe LVD, myocardial revascularization is recommended, and CABG is recommended as the primary revascularization strategy choice (class IB) by the European Society of Cardiology (ESC) and European Association for Cardio-Thoracic Surgery (EACTS) [4]. However, patients with CAD complicated by LVD, particularly where the left ventricular ejection fraction (LVEF) is less than 35%, represent a higher-risk population with specific considerations and challenges for conventional on-pump coronary artery bypass grafting (ONCAB) [5,6,7,8]. Ventricular remodeling after myocardial ischemia contributes to increased myocardial contractility in the early stage but accelerates the loss of myocardial function in the late stage [9]. The significant enlargement of the left ventricle further reduces the tolerance to surgery and increases the difficulty of exposure and anastomosis of the target vessels [10]. It has been shown that off-pump coronary artery bypass grafting (OPCAB) can reduce postoperative complications and benefit high-risk patients [11,12]. However, there is a dearth of reports on whether patients with severe left ventricular dysfunction (LVD) and a giant left ventricle can derive greater benefits from OPCAB. Therefore, the purpose of this study was to compare the short- and long-term outcomes of OPCAB and ONCAB in such patients.

## 2. Materials and Methods

### 2.1. Patients

From January 2011 to January 2021, a total of 98 patients with severe LVD (LVEF < 35%) and a giant left ventricle (LVEDD > 6.0 cm) underwent isolated CABG in Wuhan Union Hospital. Among these patients, 52 received OPCAB and 46 received ONCAB. Patients with conversion from off-pump to on-pump surgery were included into the OPCAB group. All surgeons in this study had extensive experience in both approaches over a 10- year period (more than 50 per year, respectively). The Medical Ethics Committee of Tongji Medical College, Huazhong University of Science and Technology approved the ethics of this study (IORG No. IORG0003571) and patient informed consent was waived.

### 2.2. Surgical Technique

The standard median sternum incision was performed to expose the heart in both surgical approaches. For ONABG, the cardiopulmonary bypass technique established by aortic inflow cannulation and right atrium outflow cannulation, or superior and inferior vena cava outflow cannulation, was used and myocardial protection was performed by intermittent antegrade cold blood cardioplegia. All anastomosis was performed under cardiac arrest and the CPB flow was maintained at around 2.5 L/min/m^2^. For OPCAB, commercially available cardiac positioning techniques and coronary artery stabilizers were used. The conventional anastomosis is the left anterior descending artery (LAD) with left internal mammary artery (LIMA), great saphenous vein and/or radial artery anastomosis with other target vessels in both surgical approaches. Except for differences in surgical procedures, in-hospital management for patients was similar.

### 2.3. Study Variables

The preoperative variables included sex, age, and body mass index (BMI). Additionally, concomitant medical diseases, including diabetes, hypertension, chronic renal insufficiency, renal-replacement therapy, hepatic insufficiency, peripheral arterial disease, stroke, and chronic obstructive pulmonary disease (COPD) were recorded. In this study, renal insufficiency was defined as creatinine clearance (estimated glomerular filtration rate [eGFR]) <60 mL/min and hepatic insufficiency was serum total bilirubin (TBIL) >17.1 μmol/L or aminotransferase >40 IU/L. Cardiac variables included a history of myocardial infarction, percutaneous coronary intervention (PCI), intra-aortic balloon pump (IABP), atrial fibrillation, left ventricular ejection fraction (LVEF), left ventricular end-diastolic diameter (LVEDD), and number of diseased vessels. Variables associated with revascularization included number of grafts, number of distal anastomosis and incomplete revascularization rate. The primary early outcomes included death, nonfatal myocardial infarction, nonfatal stroke, and new renal failure requiring dialysis within 30 days. Other outcomes included IABP use, low cardiac output syndrome, new-onset atrial fibrillation, respiratory failure or infection, new renal insufficiency, new hepatic insufficiency, sternum infection, and reoperation for bleeding. Hospital stay time and postoperative intensive care unit (ICU) and ventilator assistance time were also collected to assess clinical efficacy.

### 2.4. Following Up

Follow-up information was obtained through telephone and outpatient review follow-up data. In January 2021, the research staff telephoned all patients or their next of kin (if patients were not available) to obtain information on survival and events. The two long-term outcomes were death from any cause and a composite of major adverse cardiovascular events (MACE), defined as death from cardiac cause, nonfatal myocardial infarction, or repeat revascularization (CABG or PCI).

### 2.5. Statistical Analysis

All clinical data in this study were obtained from the electronic medical record system of our medical center. The method of obtaining long-term survival data has been described above. Normally distributed continuous variables were expressed as mean ± SD, and non-normally distributed continuous variables are shown as median or interquartile range. Normally distributed data were analyzed with the Student’s *t*-test, while non-normally distributed data were analyzed using the Mann–Whitney U test. Categoric variables were presented as counts with percentages and analyzed using the χ^2^ tests or Fisher’s exact test. We performed a propensity score analysis 1:1 nearest neighbor matching by a logistic regression to adjust for differences in baseline characteristics of patients in the two groups. The defined matching criteria were gender, age, BMI, smoker status, hypertension, diabetes, myocardial infarction, PCI, peripheral arterial disease, stroke, renal insufficiency, hepatic insufficiency, COPD, Aarial fibrillation, LVEF, LVEDD, IABP use, timing of surgery, extent of CAD and left main disease. Standardized mean differences of matched variables were used to evaluate the balance of the two matched groups. A maximum standardized mean difference of 0.1 is generally considered acceptable. After matching, normally distributed continuous variables were analyzed using a paired *t*-test, while non-normally distributed variables were analyzed with the Wilcoxon test. Categoric variables were analyzed using a paired chi-square test (McNemar test). A *p* value less than 0.05 was considered significant statistically. The IBM SPSS (version 23, Armonk, NY, USA) software was used to analyze data. The Kaplan–Meier survival curves and log-rank test were used to compare the difference in the long-term outcomes between the ONCAB and OPCAB group.

## 3. Results

### 3.1. Patient Characteristics

A total of 98 patients with LVEF less than 35% and a giant left ventricle who underwent isolated CABG were eligible for this study. Among them, 46 received ONCAB and 52 received OPCAB. The baseline characteristics of the patients before matched are shown in Table 1. The mean age of patients in the OPCAB group was higher than that in ONCAB (58.4 ± 14.1 vs. 52.2 ± 11.5; *p* = 0.019) and there were more patients with hypertension in the OPCAB group (73.1% vs. 47.8%; *p* = 0.010). No significant differences were observed in the other baseline characteristics. After adjustment, 44 pairs for each group were selected and the baseline characteristics of patients in the two groups were comparable (Table 2).

### 3.2. Revascularization Data and Early Outcomes

In the unadjusted study groups, the OPCAB group had less distal anastomosis (3.2 ± 0.8 vs. 3.6 ± 0.9; *p* = 0.023) and a higher rate of incomplete revascularization (25.0% vs. 8.7%; *p* = 0.033) (Table 3). After propensity matching, the number of distal anastomosis (3.2 ± 0.9 vs. 3.6 ± 1.0; *p* = 0.066) were comparable between the two groups. The OPCAB group still had a higher rate of incomplete revascularization (25.0% vs. 9.1%; *p* = 0.047) (Table 4).

In the unadjusted study groups, the risk of postoperative IABP use (9.6% vs. 26.1%; *p* = 0.032) in the OPCAB group was significantly lower than that of the ONCAB group. However, the incidence of postoperative death, myocardial infarction, stroke, and other outcomes were similar between the two groups (Table 3). After propensity matching, OPCAB still showed advantage in reducing postoperative IABP use (9.1% vs. 25.0%; *p* = 0.047). Besides, patients in the OPCAB group had a significantly lower risk of renal insufficiency (11.4% vs. 29.5%; *p* = 0.034). As with unadjusted results, no significant difference existed in the postoperative mortality, myocardial infarction, stroke, and other outcomes between the two groups (Table 4).

### 3.3. Long-Term Outcomes

**Figure 1:**
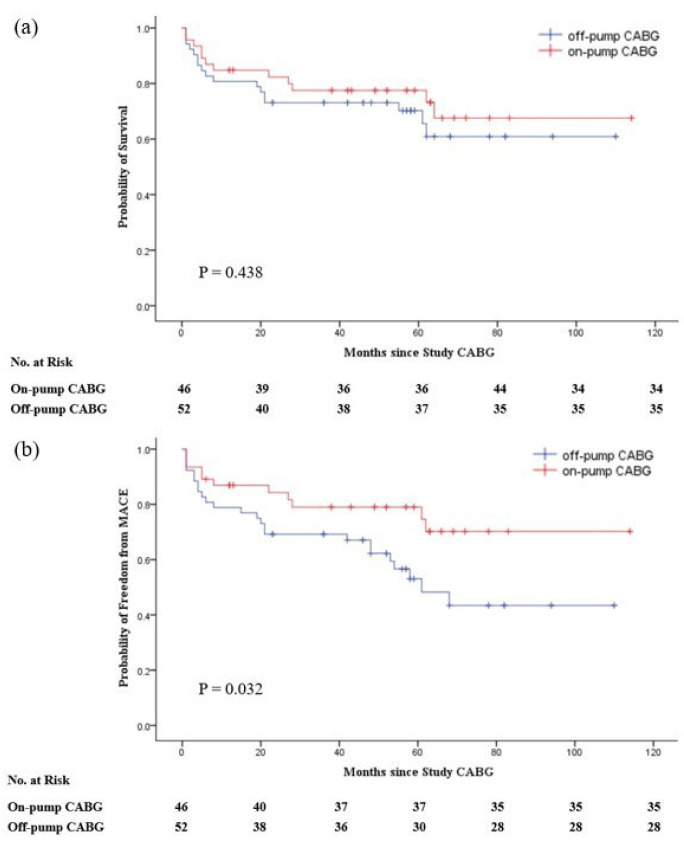
Kaplan–Meier estimates of rates of survival (**a**) and major adverse cardiovascular events (MACE) (**b**) before matched. Survival calculations were based on deaths from any cause. The composite MACE outcome was defined as death from any cause, nonfatal myocardial infarction, or repeat revascularization (CABG or PCI).

**Figure 2:**
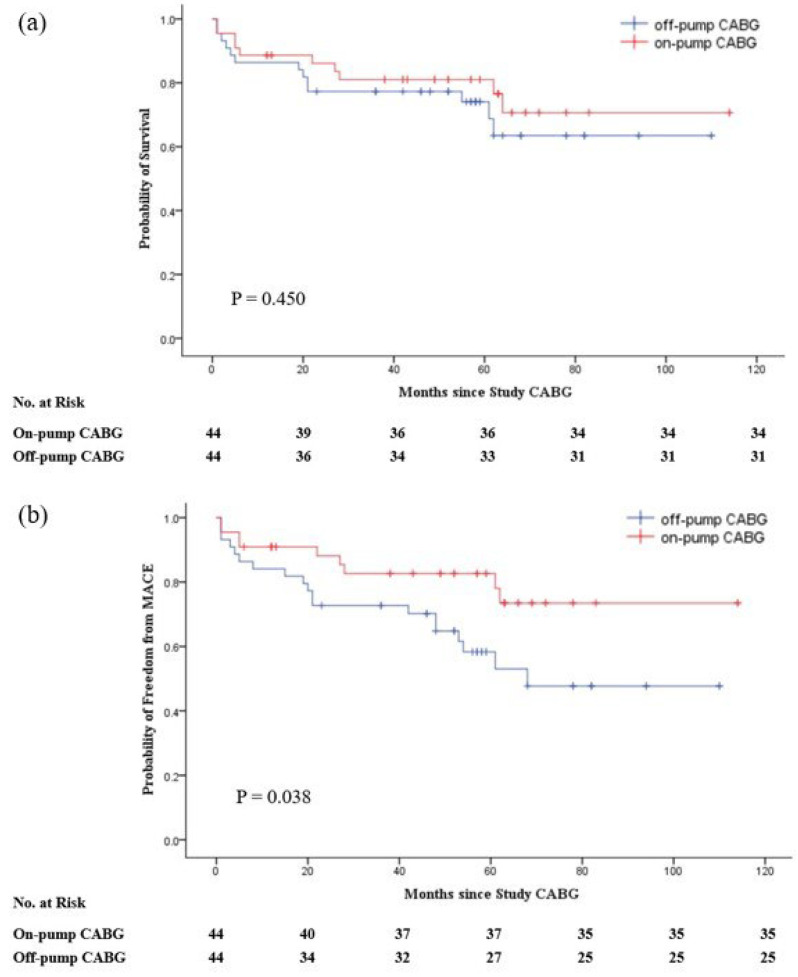
Kaplan–Meier estimates of rates of survival (**a**) and major adverse cardiovascular events (MACE) (**b**) after matched. Survival calculations were based on deaths from any cause. The composite MACE outcome was defined as death from any cause, nonfatal myocardial infarction, or repeat revascularization (CABG or PCI).

In the unadjusted study groups, the long-term survival rate in the ONCAB group was slightly higher than that in the OPCAB group, but not statistically significant (log-rank test; *p* = 0.438). The ONCAB group had a significant advantage in reducing the occurrence of MACE (log-rank test; *p* = 0.032) (Figure 1). After matching, The ONCAB group still had a slightly higher long-term survival rate (log-rank test; *p* = 0.450) and a significantly lower incidence of MACE (log-rank test; *p* = 0.038) (Figure 2).

## 4. Discussion

Our study showed that more elderly and hypertensive patients received OPCAB before matching, suggesting that clinicians prefer OPCAB for revascularization in these patients. For patients with severe LVD and a giant left ventricle, both approaches improved left ventricular function, which was reflected in an increase in LVEF and a decrease in LVEDD. The 30-day, 5-year and 10-year mortality rates in our study were lower than those in the STICH trial, which showed that the outcomes of CABG in our center are satisfactory [2,3]. Propensity score matching made the baseline characteristics of the two groups comparable, therefore, the differences in short- and long-term outcomes between the two approaches can be compared more objectively.

Considering the possibility of delayed death in high-risk patients, true early outcomes should include 1-year mortality. In our study, there was no significant difference in postoperative mortality at 30-day and 1-year between the two groups. Although the result was consistent with a meta-analysis and several previous observational and propensity-matched studies in patients with severe LVD [13,14,15], the STS National Database (SND) and Japanese Adult Cardiovascular Surgery (JACSD) studies had demonstrated that OPCAB was significantly associated with lower early postoperative mortality compared with ONCAB [16,17]. The SND study reported a higher percentage of preoperative intra-aortic balloon pump (IABP) usage in the ONCAB group although significant differences were eliminated by propensity score matching. It still suggested that more patients had hemodynamic compromise or a pre-shock state in the SND study, and were at high risk of perioperative mortality. A study of data from the STS National Database by Chawla and colleagues showed that OPCAB was associated with reduced mortality in patients with poor preoperative renal function [18]. The high proportion of chronic kidney disease (14.1% in ONCAB and 12.2% in OPCAB) could explain the strong decrease in mortality of OPCAB in the JACSD study. It should also be noted that none of the above studies provided data on left ventricle structure. Ventricular remodeling after myocardial ischemia contributes to increased myocardial contractility in the early stage but accelerates the loss of myocardial function accompanied by significant changes in cardiac diameter in the late stage [9]. A severely dilated left ventricle could increase technical challenges, especially in maintaining hemodynamic stability and lateral wall grafting during surgery [10]. These challenges are exactly what OPCAB is controversial for.

Despite the above disadvantages, our study showed that OPCAB was able to reduce early postoperative renal insufficiency and IABP use. The advantage of OPCAB may be related to the avoidance of CPB and cardiac arrest. It has been confirmed that CPB can promote the production of inflammatory mediators and activate the pathway of complement [19,20]. Rothenburger et al. also proved that CPB could induce the imbalance between inflammation and anti-inflammatory mediators, further triggering the systemic inflammatory response syndrome [21]. The kidneys are sensitive to inflammatory factors. Animal models clearly demonstrated the role of inflammation in renal tubular injury and dysfunction [22,23]. Besides, recovery from myocardial stunning and rewarming on CPB have been proven to be risk factors for acute kidney injury [24]. In patients with severe LVD and a giant left ventricle, the reserve of left ventricular function is nearly depleted. Despite the protection of cold blood cardioplegia, cardiac re-beating is still a challenge. The higher IABP usage rate also suggested that more patients receiving ONCAB had difficulty in recovering cardiac function and require the assistance of external machines. Myocardial ischemia-reperfusion injury and the inflammatory response might be the trigger for the sharp decline of cardiac function in the early postoperative period [25]. Therefore, OPCAB appears to be a safer way to achieve revascularization in such patients compared to ONCAB.

For cardiac surgery, not only safety, but also long-term benefits must be considered. Our study showed that the OPCAB group had similar long-term survival to ONCAB, but a significantly higher incidence of MACE. It is generally accepted that the higher rate of incomplete revascularization of OPCAB could impair long-term outcomes. Similar to the findings of many studies [11,12,26], more incomplete revascularization was observed in the OPCAB group. The left ventricle of the patients was enlarged significantly in this study, which made the fixation and anastomosis of the target vessel more difficult [27]. In addition, severe LVD results in significantly reduced tolerance to cardiac translocation during vascular anastomosis. Therefore, the adverse effects of incomplete revascularization of OPCAB appear to be more pronounced in patients with severe LVD and a giant left ventricle. Benedetto et al. also proved that when only one coronary territory was left ungrafted, the impact of incomplete revascularization on long-term outcomes was marginal after NOCAB but significant after OPCAB [28]. The goal of CABG is to achieve complete revascularization, based on the dogma that complete revascularization leads to better early and long-term survival [29]. The association between complete revascularization and reduced risk of subsequent cardiovascular events may be causal. Studies have shown that complete revascularization could improve clinical outcomes by reducing or eliminating myocardial ischemia, especially in the setting of greater myocardial ischemia [30]. However, some scholars have suggested that incomplete revascularization may be an alternative for higher comorbidity burden and advanced coronary artery disease not amenable to complete revascularization [27]. Our study showed that higher rates of incomplete revascularization and long-term MACE coexist in the OPCAB group, suggesting that incomplete revascularization is associated with poorer long-term outcomes in patients with severe LVD and a giant left ventricle. In order to ensure long-term quality of life, it is still necessary to achieve complete revascularization as much as possible in such patients.

It is well known that technical skill, surgical experience, and operative judgment play an important role in patient prognosis. In the ROOBY trial, OPCAB had higher postoperative 5-year mortality than ONCAB [31]. Proponents of OPCAB criticized the design of the ROOBY trial, arguing that the trial allowed surgical residents and VA surgeons who were inexperienced with OPCAB to be the operating surgeons and the associated high rate of incomplete revascularization impaired long-term outcomes [26]. When the CORONARY trials required operators to have extensive OPCAB experience, the results showed the rate of incomplete revascularization decreased in the OPCAB group and 5-year survival were similar between the two groups [32]. Notably, the latest 10-year outcomes of the ROOBY trial showed that the OPCAB group had significantly shorter median time to composite end point including death and subsequent revascularization (4.6 vs. 5.0; OPCAB and ONCAB respectively; years) [33]. The 10-year outcomes of the CORONARY trial are worth looking forward to. To exclude the bias of surgical experience on outcomes, all chief surgeons and their teams included in this study had completed more than 200 on-pump and 100 off-pump CABG, respectively, before 2011. The results suggested that OPCAB still had disadvantage in achieve complete revascularization. The OPCAB reduced early postoperative complications while increasing the risk of long-term MACE in patients with severe LVD and a giant left ventricle. The choice of OPCAB for these patients still needs to be cautious.

There are several limitations in this study. Firstly, the study was retrospective, which may confer unavoidable confounding factors. In addition, the study was a single-center study and the general applicability of the results are worth discussing. Besides, in our center, antegrade perfusion is used. If anterograde and retrograde perfusion are routinely used, it may provide better myocardial protection and improve the prognosis of ONCAB. Finally, the study had a small sample size, thus it had poor sensitivity to find differences between groups.

## 5. Conclusions

For patients with severe LVD and a giant left ventricle, both ONCAB and OPCAB approaches could improve left ventricular function. However, OPCAB reduced early postoperative complications while sacrificing long-term quality of life compared to ONCAB. The choice of OPCAB for these patients needs to be made with caution. Given that this was a single-center study, a multi-center study should be performed to obtain more generalized and realistic results.

## Figures and Tables

**Table 1:** Baseline characteristics of the patients before matching.

Characteristic	Off-Pump CABG (*n* = 52)	On-Pump CABG (*n* = 46)	*p* Value
Age—year	58.4 ± 14.1	52.2 ± 11.5	0.019
Male sex—no. (%)	40 (76.9%)	38 (82.6%)	0.486
BMI	24.1 ± 3.0	23.5 ± 2.9	0.345
Smoke	26 (50.0%)	22 (47.8%)	0.830
Clinical history—no. (%)			
Hypertension	38 (73.1%)	22 (47.8%)	0.010
Diabetes	24 (46.2%)	16 (34.8%)	0.253
Myocardial infarction	36 (69.2%)	26 (56.5%)	0.193
PCI	18 (34.6%)	18 (39.1%)	0.644
Peripheral arterial disease	10 (19.2%)	4 (8.7%)	0.137
Stroke	20 (38.5%)	18 (39.1%)	0.946
Renal insufficiency	10 (19.2%)	4 (8.7%)	0.137
Hepatic insufficiency	10 (19.2%)	6 (13.0%)	0.408
COPD	18 (34.6%)	24 (52.2%)	0.080
Atrial fibrillation	9 (17.3%)	6 (13.0%)	0.558
LVEF—%	31.3 ± 3.3	31.6 ± 2.6	0.404
LVEDD—cm	6.3 ± 0.4	6.4 ± 0.5	0.394
IABP use—no. (%)	4 (7.7%)	4 (8.7%)	1.000
Urgent surgery—no. (%)	4 (7.7%)	2 (4.3%)	0.681
Diseased vessels—no./total no. (%)			
1-Vessel	0 (0.0%)	0 (0.0%)	-
2-Vessel	8 (15.4%)	4 (8.7%)	0.313
3-Vessel	44 (84.6%)	42 (91.3%)	0.313
Mean of diseased vessels	2.8 ± 0.4	2.9 ± 0.3	0.311
Left main > 50%	16 (30.8%)	10 (21.7%)	0.312

**Table 2:** Baseline characteristics of the patients after matching.

Characteristic	Off-Pump CABG (*n* = 44)	On-Pump CABG (*n* = 44)	*p* Value
Age—year	56.9 ± 13.8	52.6 ± 11.6	0.116
Male sex—no. (%)	32 (72.7%)	36 (81.8%)	0.309
BMI	24.3 ± 3.1	23.4 ± 2.9	0.164
Smoke	25 (56.8%)	21 (47.7%)	0.393
Clinical history—no. (%)			
Hypertension	30 (68.2%)	22 (50.0%)	0.083
Diabetes	19 (43.2%)	16 (36.4%)	0.513
Myocardial infarction	31 (70.5%)	24 (54.5%)	0.123
PCI	17 (38.6%)	18 (40.9%)	0.828
Peripheral arterial disease	7 (15.9%)	4 (9.1%)	0.334
Stroke	16 (36.4%)	18 (40.9%)	0.661
Renal insufficiency	10 (22.7%)	4 (9.1%)	0.080
Hepatic insufficiency	9 (20.5%)	6 (13.6%)	0.395
COPD	18 (40.9%)	22 (50.0%)	0.392
Atrial fibrillation	8 (18.2%)	5 (11.4%)	0.367
LVEF—%	31.3 ± 3.1	31.5 ± 2.7	0.640
LVEDD—cm	6.4 ± 0.4	6.4 ± 0.5	0.550
IABP use—no. (%)	3 (6.8%)	2 (4.5%)	1.000
Urgent surgery—no. (%)	4 (9.1%)	2 (4.5%)	0.676
Diseased vessels—no./total no. (%)			
1-Vessel	0 (0.0%)	0 (0.0%)	-
2-Vessel	7 (15.9%)	3 (6.8%)	0.179
3-Vessel	37 (84.1%)	41 (93.2%)	0.179
Mean of diseased vessels	2.8 ± 0.4	2.9 ± 0.3	0.184
Left main > 50%	12 (27.3%)	10 (22.7%)	0.622

**Table 3:** Operative characteristics and early outcome of the patients before matching.

Characteristics	Off-Pump CABG (*n* = 52)	On-Pump CABG (*n* = 46)	*p* Value
**Operative characteristics**			
No. of distal anastomosis—mean	3.2 ± 0.8	3.6 ± 0.9	0.023
LIMA use—no. (%)	47 (90.4%)	41 (89.1%)	1.000
Incomplete revascularization—no. (%)	13 (25.0%)	4 (8.7%)	0.033
Ventilator assistance time—hours, mean	35.7 ± 37.1	46.2 ± 35.4	0.156
Postoperative ICU stay—days, mean	3.1 ± 4.2	4.7 ± 7.7	0.200
Hospital stay time—days, mean	28.4 ± 8.3	30.4 ± 9.7	0.713
Mean of postoperative LVEF	42.9 ± 8.2	41.3 ± 9.9	0.733
Mean of postoperative LVEDD	5.6 ± 0.6	5.8 ± 0.8	0.189
**Early outcome**			
Primary outcomes—no. (%)			
Death	3 (5.8%)	2 (4.3%)	1.000
Myocardial infarction	3 (5.8%)	3 (6.5%)	1.000
Stroke	4 (7.7%)	4 (8.7%)	1.000
New renal failure requiring dialysis	2 (3.8%)	6 (13.0%)	0.142
Other outcomes—no. (%)			
New-onset atrial fibrillation	8 (15.4%)	12 (26.1%)	0.190
Low cardiac output syndrome	14 (26.9%)	18 (39.1%)	0.198
IABP use	5 (9.6%)	12 (26.1%)	0.032
Respiratory failure or infection	6 (11.5%)	6 (13.0%)	0.821
Renal insufficiency	7 (13.5%)	13 (28.3%)	0.070
Hepatic insufficiency	10 (19.2%)	12 (26.1%)	0.417
Reoperation for bleeding	1 (1.9%)	4 (8.7%)	0.183
Sternum Infection	0 (0.0%)	2 (4.3%)	0.218

**Table 4:** Operative characteristics and early outcome of the patients after matching.

Characteristics	Off-Pump CABG (*n* = 44)	On-Pump CABG (*n* = 44)	*p* Value
**Operative characteristics**			
No. of distal anastomosis—mean	3.2 ± 0.9	3.6 ± 1.0	0.080
LIMA use—no. (%)	41 (93.2%)	40 (90.9%)	1.000
Incomplete revascularization—no. (%)	11 (25.0%)	4 (9.1%)	0.047
Ventilator assistance time—hours, mean	36.7 ± 39.2	47.1 ± 35.9	0.199
Postoperative ICU stay—days, mean	3.1 ± 4.4	4.4 ± 7.8	0.358
Hospital stay time—days, mean	29.6 ± 23.0	28.8. ± 15.1	0.852
Mean of postoperative LVEF	42.0 ± 8.6	41.3 ± 10.1	0.742
Mean of postoperative LVEDD	5.7 ± 0.6	5.8 ± 0.8	0.253
**Early outcome**			
Primary outcomes—no. (%)			
Death	2 (4.5%)	2 (4.5%)	1.000
Myocardial infarction	3 (6.8%)	4 (9.1%)	1.000
Stroke	3 (4.5%)	3 (4.5%)	1.000
New renal failure requiring dialysis	2 (4.5%)	5 (11.4%)	0.434
Other outcomes—no. (%)			
New-onset atrial fibrillation	7 (15.9%)	12 (27.3%)	0.195
Low cardiac output syndrome	13 (29.5%)	17 (38.6%)	0.368
IABP use	4 (9.1%)	11 (25.0%)	0.047
Respiratory failure or infection	4 (9.1%)	6 (13.6%)	0.502
Renal insufficiency	5 (11.4%)	13 (29.5%)	0.034
Hepatic insufficiency	9 (20.5%)	12 (27.3%)	0.453
Reoperation for bleeding	1 (2.3%)	4 (9.1%)	0.360
Sternum Infection	0 (0.0%)	2 (4.5%)	0.494

## Data Availability

The raw data is available by contacting with the corresponding authors.

## Abbreviations

CABG	coronary artery bypass grafting
OPCAB	off-pump CABG
ONCAB	on-pump CABG
LVD	left ventricular dysfunction
LVEF	left ventricular ejection fraction
LVEDD	left ventricular end-diastolic diameter
LCOS	low cardiac output syndrome
MACE	major adverse cardiovascular events
CAD	coronary heart disease
PCI	percutaneous coronary intervention
CPB	cardiopulmonary bypass
LIMA	left internal mammary artery
BMI	body mass index
COPD	chronic obstructive pulmonary disease
IABP	intra-aortic balloon pump
ICU	intensive care unit
